# How COVID-19 Affected Sleep Talking Episodes, Sleep and Dreams?

**DOI:** 10.3390/brainsci14050486

**Published:** 2024-05-11

**Authors:** Milena Camaioni, Serena Scarpelli, Valentina Alfonsi, Maurizio Gorgoni, Rossana Calzolari, Mina De Bartolo, Anastasia Mangiaruga, Alessandro Couyoumdjian, Luigi De Gennaro

**Affiliations:** 1 Department of Psychology, Sapienza University of Rome, Via dei Marsi 78, 00185 Rome, Italy; milena.camaioni@uniroma1.it (M.C.); serena.scarpelli@uniroma1.it (S.S.); valentina.alfonsi@uniroma1.it (V.A.); maurizio.gorgoni@uniroma1.it (M.G.); debartolo.1847715@studenti.uniroma1.it (M.D.B.); alessandro.couyoumdjian@uniroma1.it (A.C.); 2 Body and Action Lab, IRCCS Fondazione Santa Lucia, Via Ardeatina 306, 00179 Rome, Italy; 3 Department of General Psychology, University of Padova, Via Venezia 8, 35131 Padova, Italy

**Keywords:** sleep talking, parasomnia, dreaming

## Abstract

Background: The COVID-19 pandemic increased symptoms of stress and anxiety and induced changes in sleep quality, dream activity, and parasomnia episodes. It has been shown that stressful factors and/or bad sleep habits can affect parasomnia behaviors. However, investigations on how COVID-19 has affected sleep, dreams, and episode frequency in parasomnias are rare. The current study focuses on the impact of the pandemic on a specific parasomnia characterized by speech production (sleep talking, ST). Methods: We selected 27 participants with frequent ST episodes (STs) during the pandemic and compared them with 27 participants with frequent STs from a previous study conducted during a pre-pandemic period. All participants performed home monitoring through sleep logs and recorded their nocturnal STs for one week. Results: We observed a higher frequency of STs in the pandemic group. Moreover, STs were related to the emotional intensity of dreams, independent of the pandemic condition. The pandemic was associated with lower bizarreness of dreams in the pandemic group. There were no differences in sleep variables between the two groups. Conclusion: Overall, these results suggest a stressful effect of COVID-19 on the frequency of STs. Both the pandemic and the frequency of STs affect qualitative characteristics of dreams in this population.

## 1. Introduction

The strong countermeasures to control the COVID-19 pandemic have had a profound impact on sleep and mental health worldwide, as evidenced by numerous studies [1,2,3,4]. Specifically, empirical evidence in Italy showed poor sleep quality, increased time spent in bed, and delayed sleep schedules [3,5,6]. Moreover, some studies have reported high levels of stress, depression, and anxiety symptoms [7,8,9,10].

However, the effects of these restrictions are complex. In fact, research has shown a mixed picture, with some studies reporting both beneficial and destructive effects on sleep [5,6,11,12,13,14]. On the one hand, stress and environmental circumstances of staying at home, such as reduced exposure to natural light and physical activity and increased screen exposure [15], led to increased sleep difficulties. On the other hand, individuals were able to adapt their sleep patterns to their natural circadian tendencies, resulting in longer sleep and better daytime functioning [6].

Interestingly, longitudinal studies across the different COVID-19 waves have pointed to a general improvement in sleep disturbances, depression, and anxiety symptoms during the post-lockdown period [6,16,17]. Specifically, some studies have found a high percentage of poor sleepers (51%) in the general Italian population during the initial lockdown phase, followed by a partial reversal in the subsequent follow-up phases (44% poor sleepers) [6]. This dynamic pattern of sleep changes underscores the evolving nature of the pandemic’s impact on sleep and mental health.

The COVID-19 pandemic not only affected sleep patterns but also led to significant changes in dream activity [18,19]. Studies comparing the pre-pandemic period with the lockdown phases of the first wave of the pandemic have revealed an increase in dream recall frequency in both healthy adults [18,19,20,21,22] and COVID-19 patients [23]. Additionally, dream contents were characterized by greater emotional intensity with more negative valence [20,24] compared to the pre-pandemic period. However, longitudinal studies have shown variations in dream activity and emotional features throughout the different waves of the pandemic [18], with some studies reporting a reduction in dream recall and emotional oneiric features during the second and third waves compared to the initial phase [25,26].

An additional factor of interest is the increase in nightmares observed during the pandemic [26,27]. A higher frequency of nightmares was reported during the first wave than in the pre-pandemic period, which decreased during the third wave [21,26,27,28]. This increase was associated with poor sleep and psychological symptoms (i.e., anxiety) [21,28]. In the parasomnia literature, it has been observed that stress or fragmented sleep can increase the frequency of nightmares or other altered nighttime sleep disorders [29]. 

Some studies have also shown the impact of COVID-19 on other parasomnias. In particular, the International COVID-19 Sleep Study (ICOSS) collaborative study showed a strikingly high prevalence of dream enactment behaviors (DEBs) in the general population during the pandemic [30]. Moreover, sleep phenomena such as sleep talking (ST), sleep maintenance problems, and symptoms of REM Behavior Disorder (RBD) were associated with high dream recall during the pandemic [30]. These findings are consistent with the arousal–retrieval model [31], supporting the notion that frequent arousals during sleep facilitate memory storage of dream content [19,32].

However, it remains unclear to what extent COVID-19 affects the frequency of parasomniac episodes and whether it generates a change in sleep and dreams. Studies have focused mainly on nightmares, with little investigation into other kinds of altered nighttime behaviors.

Our research aims to expand knowledge on this topic considering a specific parasomnia: sleep talking (ST), or somniloquy. ST is a common parasomnia in the general population, characterized by the production of linguistic vocalizations that can occur during all stages of sleep [33,34,35]. A recent study showed an association between verbal episodes and increased sleep fragmentation [35]. In addition, ST also appears to influence some dream characteristics, such as emotional intensity [35]. It has been also observed that sleep talking is often associated with other sleep disorders, psychiatric or medical conditions, and stress [36,37]. A recent study on young adults who self-identified as habitual sleep talkers showed a frequent co-occurrence of ST with other parasomnias, including nightmares (94%) and RBD (47%) [34]. According to this finding, we hypothesized that COVID may have also affected somniloquy in its isolated form.

Our study takes a unique approach by comparing two groups of sleep talkers recorded during the pre-pandemic and pandemic periods. This comparative analysis aims to identify differences in the frequency of the phenomenon. In addition, we investigated whether changes in oneiric characteristics could be explained by variables related to the pandemic or the parasomniac phenomenon, providing a comprehensive understanding of the topic.

## 2. Materials and Methods

### 2.1. Participants

We selected two groups of participants with frequent ST episodes (verbal activations—STs), paired by gender and age: the pandemic group (PN) (N *n* = 27; F = 21, mean age: 23.63, sd: ±2.83) and the pre-pandemic group (pre-PN) (*n* = 27; F = 21, mean age: 23.74, sd: ±2.85).

The PN was selected from a study conducted during the pandemic from January 2021 to October 2021 [35]. Meanwhile, the pre-PN was identified in a previous study performed during a pre-pandemic period, specifically from 2016 to 2018 [34].

**Figure 1:**
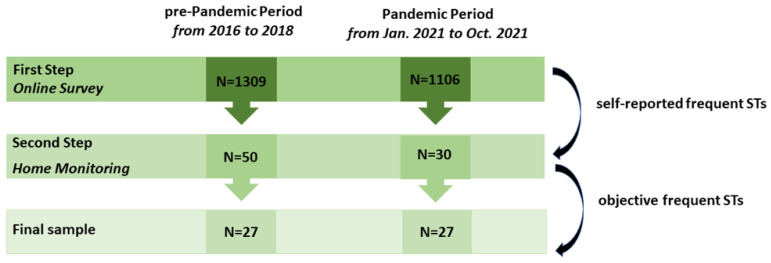
The recruitment process of the two groups.

Both groups were recruited using an identical procedure consisting of two steps (Figure 1): an online survey and home monitoring. An online survey was administered to the general population via digital platforms (e.g., Facebook, WhatsApp, Instagram). Subjects who reported frequent sleep talk were selected on the basis of this survey and were contacted to participate in home monitoring to verify the actual presence of nighttime speech. The two selection steps are discussed in detail in the following sections.

#### 2.1.1. Online Survey

The online survey consisted of an ad hoc questionnaire of case history to assess general health, the Pittsburgh Sleep Quality Index (PSQI) [38], and the Munich Parasomnia Questionnaire (MUPS) [39].

The PSQI consists of 19 self-report items that measure subjective sleep quality. The questionnaire produces a global score ranging from 0 to 21. A global score greater than 5 indicates poor sleep quality [38].

The MUPS is a 22-item self-report questionnaire assessing the presence and frequency of altered nocturnal behaviors on a Likert scale from 1 (“Never”) to 7 (“Very frequently—every or nearly every night”) [39]. We considered very frequent behaviors to be those that received a score greater than 5.

For the first step, the inclusion criteria were:

1. Participants who self-reported frequent STs, as indicated by the MUPS item related to STs (“How often do you experience the following behaviors? talking during sleep”) ranging from 5 (“Sometimes—one or more times per month) to 7 (“Very frequently—every or nearly every night”);
2. Age range: 18–35 years;
3. Absence of medical conditions and psychiatric disorders (this was investigated with a specific question in the questionnaire on case history: “Do you have acute or chronic health problems (medical or psychological problems? (If yes, please specify in ‘other’)”);
4. Absence of other sleep disorders except ST;
5. No drug or alcohol abuse.

During the pre-pandemic period, 1309 subjects responded to the online survey. From this sample, *n* = 50 met the inclusion criteria of the first phase and were recruited for home monitoring. Similarly, of the 1106 questionnaires collected during the pandemic period, *n* = 30 subjects were eligible for home monitoring (Figure 1).

#### 2.1.2. Home Monitoring

In both groups, the participants selected in the first step performed 7 days of home monitoring (see below in the procedure section).

Although the selected participants had subjectively reported a high ST frequency on the MUPS item, we wanted to ensure that they were objectively frequent sleep talkers. Therefore, only subjects who produced at least one ST episode during home monitoring were included in the final sample.

Thus, the sample selected for analysis consisted of *n* = 27 STs in the pre-PN and *n* = 27 STs in the PN.

Participants were informed of the procedure and signed a written informed consent. The protocol complied with the Declaration of Helsinki and was approved by the Institutional Review Board of the Department of Psychology of Sapienza University of Rome (protocol number of 04/02/2020: 0000226).

### 2.2. Procedure

The home monitoring procedure was the same for both groups. Participants used an open-source voice-activated recording app installed on their smartphones (Dream Talk Recorder or Voice Activated Recorder, based on iOS or Android operating system, respectively) during sleep to record ST episodes. The application activates when it detects environmental sounds above a certain threshold (e.g., sleep talking). It records for the duration of the sound and deactivates when the sound stops. A sensitivity threshold of “medium” was set by the participants. Moreover, we instructed participants to turn on the app when they went to bed and to place the recorder near the bed every night for one week. After their last awakening in the morning, participants turned off the app. Moreover, we trained participants to audio-record each recalled mental activity as accurately as possible, distinguishing when they recalled more than one dream report, and fill out sleep diaries [40] within a maximum of 15 min after awakening. Audio recordings of dream content ensure high compliance and more accurate reports [41]. We checked to see if there were other roommates or bed partners (this was investigated with a specific question in the questionnaire on case history: “Do you sleep alone or share your bed/room with another person? (a) By yourself; (b) With another person”). This revealed that 14 sleep talkers in the pre-pandemic group and 11 in the pandemic group shared a bed or room. To make sure that the recordings were carried out by the subjects themselves, we asked them to hold the phone close to the pillow on their side. This made it possible to distinguish any sounds made by other people due to proximity to the phone.

### 2.3. Measures

#### 2.3.1. Sleep Diaries

The subjective assessment of night’s sleep was obtained by sleep diaries (see [35]). The following variables were extracted:

- Sleep onset latency (SOL): the time (in minutes) it takes to fall asleep after turning off the light;
- Number of nocturnal awakenings (NOA);
- Intra-night wakefulness (ISW): subjective duration of wakefulness (in minutes) from falling asleep to the final awakening;
- Total sleep time in minutes (TST): the amount of time spent asleep;
- Total bed time (TBT): the amount of time from lights off to the final awakening;
- Sleep efficiency (SE = TST/TBT × 100).

Moreover, the sleep diaries included three items, rated on a five-point Likert scale, representing a direct judgment of sleep quality from the subjects:

- Sleep depth (1 = very light sleep, 5 = very deep sleep)
- Sleep quiet (1 = very disturbed sleep, 5 = very quiet sleep)
- Sleep restless (1 = very low-rest sleep, 5 = very high-rest sleep)

#### 2.3.2. Dream Reports

Two independent judges (R.C. and M.B.) evaluated the dream reports of each subject. The judges transcribed and counted the number of dream recalls (DRs). They then performed the “pruning” (removal of all repetitions and subjects’ inferences) to compute the total number of words (total word count—TWC). Then, they rated three qualitative variables of dreams on a Likert scale from one to six points [42]:

- Emotional intensity—EL: 1 = very low emotional intensity; 6 = very high emotional intensity;
- Vividness—VV: 1 = no image, just thinking about objects; 2 = very vague; 3 = less vague; 4 = moderately clear and vivid; 5 = clear and reasonably vivid; 6 = clear and vivid as normal vision;
- Bizarreness—B: 1 = scenes/thoughts closely related to everyday life, something that belongs to the individual’s life or to current reality, is plausible; 2 = belongs to reality but not to the life of the individual, seems unusual, strange, or illogical; 3 = plot discontinuous, changes in setting, slightly inappropriate roles; 4 = plot highly discontinuous, unlikely elements and particular settings appear, inappropriate roles; 5 = abundance of unlikely elements, settings with imaginary elements, metamorphoses, imaginary characters; 6 = impossible settings, fantastic elements and characters (with unlikely characteristics), illogicality of the plot.

The two judges resolved any discordance by consensus, and the inter-rater reliability of each scale was substantial (Cohen’s K > 70).

#### 2.3.3. Sleep Talking Episodes

Two experimenters (R.C. and M.B.) assessed, transcribed, and counted ST episodes for each subject by listening to the audio records.

### 2.4. Statistical Analyses

The statistical procedures were carried out using the Statistical Package for Social Sciences (SPSS) version 25.0.

In order to verify the absence of differences between the two groups in MUPS, the variables were computed with a Chi-square test for independence and Fisher’s exact test (FET) when appropriate by grouping the Likert scale scores into three categories: (I) absence/very low frequency (1–2), (II) low frequency (3–4), and (III) high frequency (5–7).

The PSQI global score was compared using Student’s *t*-test for independent samples.

The normal distribution of the original data was checked and skewed data distributions (i.e., SOL, NOA, ISW, DR, TWC, EL, and STs) were transformed into normal distribution using log_10_ transformation.

The number of STs, the variables of sleep logs, and dream variables collected each day during weekly home monitoring were averaged for each participant.

To assess the effect of COVID-19 on ST episodes, we performed a one-way ANOVA with GROUP (PN, pre-PN) as the between factor and STs as dependent variable.

We also performed a one-way multivariate analysis of variance (MANOVA), with GROUP (PN, pre-PN) as a between factor and sleep measures and DR as dependent variables. The dependent variables considered were SOL, NOA, ISW, TST, TBT, SE, sleepiness, depth, quiet, restlessness, and DR. The MANOVA agreed to observe differences in sleep variables and DR frequency between the two groups.

Finally, we investigated whether the pandemic scenario, ST, or sleep fragmentation (ISW) could predict dream variables. Therefore, we performed multiple linear regressions considering GROUP, STs, and ISW as independent variables to assess the best explanatory variables of the dream measures (TWC, EL, VV, B). We checked for multicollinearity between the predictors by calculating variance inflation factors (VIF < 3) before performing the regression.

## 3. Results

### 3.1. Preliminary Questionnaire

Chi-square results show that PN and pre-PN did not differ in terms of the MUPS variables (Table 1). Similarly, the PSQI global score was not significantly different between the two groups, as shown in Table 1.

### 3.2. Home Monitoring

Table 2 reports the descriptive statistics of sleep measures, dreams variables, and STs. About 54 participants, 48 sleep talkers (pandemic group, *n* = 26 (96.30%); pre-pandemic group, *n* = 22 (82.48%)) recalled at least one dream report.

**Figure 2:**
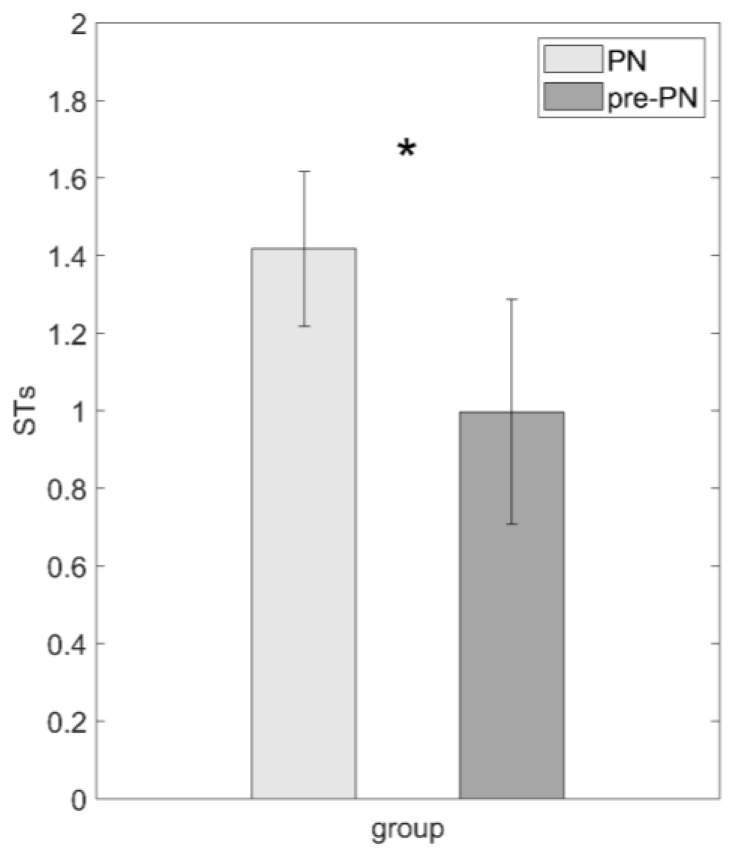
Results of the comparison between the pandemic group and pre-pandemic group. * indicates a statistical significant difference. Values are reported as raw frequencies.

PN showed a significantly higher weekly ST frequency (mean [SE] PN = 0.17 [±0.7] pre-PN = −0.27 [±0.9]) than the pre-PN group (F = 6.156; *p* = 0.016) (Figure 2).

The MANOVA performed on sleep log measures and dream frequency showed no significant differences between PN and pre-PN (Wilks’ λ = 0.786, F_10,43_ = 1.172, *p* = 0.335; η_p_^2^ = 0.214).

As shown in Table 3, the regression models were statistically significant for the EL and B dream variables (EL: adjusted R^2^ = 0.129, *p* < 0.05; B: adjusted R^2^ = 0.163, *p* < 0.05). Specifically, partial correlations show that higher frequency of STs (β = −0.393, t = −2.626, *p* = 0.012) predicted lower EL in dream reports, and the pre-pandemic group (β = −0.395, t = −2.673, *p* = 0.011) presented higher B in dream content.

## 4. Discussion

Our study aimed to investigate the phenomenon of ST during the pandemic. The results revealed a significant increase in the occurrence of STs in a group of sleep talkers monitored during the pandemic compared to a group of sleep talkers monitored in a pre-pandemic period.

Consistent with our findings, studies on parasomnias during the pandemic have shown an increase in the number of parasomnia episodes. In particular, the general population showed higher episodes of nightmares, especially in women and people with sleep disturbances, symptoms of anxiety or depression, and stress associated with COVID-19 [21,22,24,28,43]. Similarly, the International COVID-19 Sleep Study observed a higher prevalence of dream-enacting behavior in the general population than previous epidemiologic studies [30]. DEB has been associated with some factors, such as lifestyle, post-traumatic stress disorder (PTSD), depression, and anxiety symptoms [30].

Previous studies have shown that some stressful factors and poor sleep hygiene could facilitate the onset of parasomnia episodes [29]. For instance, nightmares are often associated with PTSD [44,45,46,47,48] and are reactive to stressful life events [49,50,51,52]. Moreover, several studies have found that sleep paralysis may be facilitated by stress, PTSD, and irregular sleep–wake schedules [53,54,55,56]. Finally, episodes of NREM parasomnias appear to be triggered by sleep-disrupting factors, and many clinical observations support the hypothesis that stress and anxiety are related to the frequency of episodes, although evidence in the literature is sparse [57].

Along this vein, symptoms of anxiety, depression, and impaired sleep quality during the COVID-19 pandemic [3,9,58,59] may be associated with high levels of distress, which may facilitate parasomnia episodes [60], including ST. It has been observed that ST is associated with more disturbed sleep and altered macrostructure than healthy subjects [34,35]. It is hypothesized that there is a sensitivity in the sleep talker population when important changes occur that may alter sleep patterns and affect stress and mood, promoting a greater susceptibility to the onset of parasomnia.

The current study did not find worse sleep during the pandemic period. It must be considered that the pandemic group was recorded for an extended period after the second wave, during which the pandemic restrictions underwent several changes. Sleep quality in the pandemic group may have been improved compared to the first wave and partially restored, more closely resembling the pre-pandemic period [6,16,17].

Regarding dream activity, the regression model indicates that a higher frequency of ST episodes predicted lower emotional intensity. This result suggests that dreams of sleep talkers may be characterized by lower emotional intensity, independent of the pandemic state. As suggested by a previous study, low EL may reflect emotional regulation processes [35]. Dream-related emotional regulation attenuates the emotional intensity of waking activity [61,62]. Moreover, ST has been proposed as a dream-enactment behavior that may provide direct access to cognitive processes during sleep [33,34,35], and STs are often characterized by emotional content [33,63]. Therefore, STs may reflect the mechanism of dreams in the elaboration of emotional experiences.

An alternative explanation is that the low emotional intensity may represent an intrinsic feature of sleep talkers’ dreams. Different parasomnias may manifest qualitative differences in dream activity [64,65,66]. Studies analyzing the qualitative characteristics of sleep mentation in RBD and disorders of arousals (DOAs) report that dream content associated with DEB episodes, collected in the morning after overnight laboratory monitoring, was less frequently recalled in RBD and DOAs [66]. Moreover, sleepwalkers recalled more vivid and complex plots than RBD patients, whereas bizarreness did not differ between the two groups [66].

In contrast, the finding on bizarreness is not consistent with the literature on dream activity during COVID-19. To date, the qualitative characteristics of dreams in ST are poorly investigated. Dream features of parasomnias could be due to the sleep stage from which the dream experience is collected (e.g., REM sleep in RBD, NREM sleep in somnambulism), as dream reports differ between REM-associated (dreamlike: more emotional, vivid, and bizarre mentation) and NREM-associated (thought-like: less emotional intensity and more realistic content) [67,68,69,70]. ST can occur during all sleep phases, although more frequently during NREM sleep periods [34,71,72], especially during N2 [34]. Moreover, the alteration in macrostructure in ST showed a lower amount of REM sleep and a higher amount of NREM sleep [34]. From this perspective, we speculatively suggest that a high frequency of ST could influence sleep architecture, which may have resulted in greater dream recall from NREM and lower bizarreness in the pandemic group. On the other hand, we should consider the possibility that the two groups may have basic and uncontrolled differences that explain this result.

### Limitations

One of the most critical limitations of the study is its cross-sectional rather than longitudinal design. We cannot rule out the possibility that subjects in the two groups had prior differences in the frequency of episodes. The pandemic group was also recorded over a long period characterized by different phases of the pandemic trend, which may have influenced the results. In addition, the online survey did not investigate the presence or absence of previous COVID-19 infection.

Moreover, we can only indirectly hypothesize that stressful and emotional factors related to COVID-19 affected the production of STs because direct measures of daytime emotions were not collected.

Additional limitations include the small sample size, which does not allow generalization to the population of sleep talkers, and the lack of objective sleep measures. Instrumental measures (i.e., polysomnography) would have allowed for analysis of micro- and macrostructural sleep and EEG features of dreaming.

Finally, it should be considered that one of the advantages of the study is that sleep talkers were recorded in an ecological a organic environment. However, it is important to consider the intrinsic limitations of the voice-activated application. It may not have been able to capture all ST episodes, and it may not have been able to distinguish the sound produced by other individuals sharing the bed or room with the experimental subjects.

## 5. Conclusions

To the best of our knowledge, the present study was the first to investigate the phenomenon of sleep talking during the pandemic compared to a pre-pandemic period.

The results show an increase in ST episodes during the pandemic, which may have been due to the negative impact of COVID-19 on sleep and mood. We hypothesize a sensitivity of sleep talkers to high levels of stress or changes in mood tone, which would facilitate the occurrence of ST.

Furthermore, both the pandemic scenario and the frequency of STs affected the dream contents of sleep talkers.

Overall, understanding which factors may determine the frequency of parasomniac episodes may shed light on the etiology of the phenomenon. In addition, it would be possible to define treatment protocols when the phenomenon is disruptive.

## Figures and Tables

**Table 1:** Results of comparisons of measures derived from the Munich Parasomnia Questionnaire (MUPS) and the Pittsburgh Sleep Quality Index (PSQI).

Preliminary Questionnaires								
MUPS	Pandemic (%)			Pre-Pandemic (%)			Fisher’s Exact	*p*
	I	II	III	I	II	III		
**Hypnic jerks**	11.10	33.30	55.60	11.10	22.20	66.70	0.95	0.73
**Rhythmic foot movements**	59.30	18.50	22.20	40.70	14.80	44.40	3.02	0.28
**Rhythmic movement disorders**	92.60	7.40	0.00	81.50	11.10	7.40	2.08	0.50
**Exploding head syndrome**	74.10	14.80	11.10	85.20	7.40	7.40	1.13	0.62
**Hypnagogic hallucinations**	63.00	25.90	11.10	77.80	14.80	7.40	1.48	0.56
**Periodic leg movements**	81.50	11.10	7.40	70.40	18.50	11.10	0.99	0.65
**Nocturnal leg cramps**	55.60	37.00	7.40	59.30	29.60	11.10	0.54	0.85
**Sleep-related bruxism**	63.00	7.40	29.60	66.70	3.70	29.60	0.48	1.00
**Abnormal swallowing**	81.50	7.40	11.10	85.20	11.10	3.70	1.22	0.75
**Groaning**	48.10	14.80	37.00	55.60	18.50	25.90	0.83	0.76
**Sleep enuresis**	96.30	3.70	0.00	96.30	3.70	0.00		1.00
**Nightmares**	3.70	37.00	59.30	3.70	48.10	48,10	0.95	0.79
**Sleep terrors**	70.40	14.80	14.80	51.90	29.60	18.50	2.19	0.35
**Nocturnal eating**	100.00	0.00	0.00	88.90	7.40	3.70	2.74	0.24
**Sleep-related eating**	92.60	7.40	0.00	96.30	3.70	0.00		1.00
**Confusing arousals**	59.30	25.90	14.80	59.30	22.20	18.50	0.27	1.00
**Sleep paralysis**	85.20	7.40	7.40	88.90	7.40	3.70	0.56	1.00
**Sleepwalking**	88.90	11.10	0.00	77.80	14.80	7.40	2.02	0.39
**Violent behavior**	77.80	18.50	3.70	85.20	11.10	3.70	0.84	0.85
**RBD**	55.60	33.30	11.10	51.90	33.30	14.80	0.27	1.00
**Others**	88.90	7.40	3.70	66.70	18.50	14.80	3.71	0.18
**PSQI**	**Pandemic**			**Pre-Pandemic**			***t***	***p***
**Global Score**	5.85 (0.55)			6.04 (0.52)			−0.25	0.80

MUPS, Munich Parasomnia Questionnaire; RBD, REM Behavior Disorder; PSQI, Pittsburgh Sleep Quality Index.

**Table 2:** Sleep and dream measures in the two groups.

Variables		Pandemic Mean (sd)	Pre-Pandemic Mean (sd)
STs *		1.417 (1.06)	0.996 (1.51)
**Sleep variables**	SOL *	14.087 (10.22)	10.102 (4.09)
	NOA *	1.218 (0.79)	1.593 (1.37)
	ISW *	4.320 (3.23)	6.709 (7.74)
	**TBT**	493.330 (49.14)	503.367 (76.60)
	**TST**	441.661 (36.90)	457.102 (64.07)
	**SE (%)**	90.040 (4.38)	90.930 (5.19)
	**Sleep depth**	3.787 (0.58)	3.73 (0.58)
	**Sleep quiet**	3.371 (0.63)	3.312 (0.72)
	**Sleep restless**	3.453 (0.59)	3.279 (0.56)
**Dream variables**	DR frequency *	0.701 (0.52)	0.599 (0.69)
	TWC *	73.059 (64.14)	124.819 (88.89)
	EL *	2.429(1.12)	2.767 (0.88)
	**B**	1.903 (0.92)	2.750 (0.86)
	**VV**	2.972 (0.82)	3.582 (0.90)

* Values are reported as raw data. STs, sleep talking episodes; SOL, sleep onset latency; NOA, number of nocturnal awakenings; ISW, intra-sleep wakefulness; TBT, total bed time; TST, total sleep time; SE, sleep efficiency; DR, dream recall; TWC, total word count; EL, emotional intensity; B, bizarreness; VV, vividness.

**Table 3:** The multiple linear regression models.

Dependent Variables	Predictors	Standardized β Coefficients	*t*	*p*
**TWC**				
Adjusted R^2^ = 0.087	Group	−0.289	−1.869	0.068
F = 2.488	STs	−0.166	−1.082	0.285
*p* = 0.073	ISW	0.019	0.128	0.899
**EL**				
Adjusted R^2^ = 0.129	Group	−0.090	−0.599	0.553
F = 3.327	STs	−0.393	−2.626	0.012 *
*p* = 0.028	ISW	0.030	0.208	0.836
**B**				
Adjusted R^2^ = 0.163	Group	−0.395	−2.673	0.011 *
F = 4.057	STs	−0.018	−0.124	0.902
*p* = 0.012	ISW	0.171	1.217	0.230
**VV**				
Adjusted R^2^ = 0.056	Group	−0.336	−2.138	0.038
F = 1.928	STs	−0.006	−0.037	0.971
*p* = 0.139	ISW	0.015	0.097	0.923

STs, sleep talking episodes; ISW, intra-sleep wakefulness; TWC, total word count; EL, emotional intensity; B, bizarreness; VV, vividness. * indicates statistical significance

## Data Availability

The data presented in this study are available on request to the corresponding author. The data are not publicly available due to legal and ethical reasons.

## References

[1] Jahrami H., BaHammam A.S., Bragazzi N.L., Saif Z., Faris M., Vitiello M.V. (2021). Sleep Problems during the COVID-19 Pandemic by Population: A Systematic Review and Meta-Analysis. J. Clin. Sleep Med..

[2] Rajkumar R.P. (2020). COVID-19 and Mental Health: A Review of the Existing Literature. Asian J. Psychiatr..

[3] Salfi F., Amicucci G., Cascioli J., Corigliano D., Viselli L., Tempesta D., Ferrara M. (2020). The Impact of Home Confinement Due to COVID-19 Pandemic on Sleep Quality and Insomnia Symptoms among the Italian Population. https://www.researchgate.net/profile/Federico-Salfi/publication/344337922_The_impact_of_home_confinement_due_to_COVID-19_pandemic_on_sleep_quality_and_insomnia_symptoms_among_the_Italian_population/links/5f69c99392851c14bc8e03c4/The-impact-of-home-confinement-due-to-COVID-19-pandemic-on-sleep-quality-and-insomnia-symptoms-among-the-Italian-population.pdf.

[4] Cao W., Fang Z., Hou G., Han M., Xu X., Dong J., Zheng J. (2020). The Psychological Impact of the COVID-19 Epidemic on College Students in China. Psychiatry Res..

[5] Casagrande M., Favieri F., Tambelli R., Forte G. (2020). The Enemy Who Sealed the World: Effects Quarantine Due to the COVID-19 on Sleep Quality, Anxiety, and Psychological Distress in the Italian Population. Sleep Med..

[6] Alfonsi V., Gorgoni M., Scarpelli S., Zivi P., Sdoia S., Mari E., Fraschetti A., Ferlazzo F., Giannini A.M., De Gennaro L. (2021). COVID-19 Lockdown and Poor Sleep Quality: Not the Whole Story. J. Sleep Res..

[7] Mazza C., Ricci E., Colasanti M., Ferracuti S., Napoli C., Roma P. (2020). A Nationwide Survey of Psychological Distress among Italian People during the COVID-19 Pandemic: Immediate Psychological Responses and Associated Factors. Int. J. Environ. Res. Public Health.

[8] Moccia L., Janiri D., Pepe M., Dattoli L., Molinaro M., De Martin V., Chieffo D., Janiri L., Fiorillo A., Sani G. (2020). Affective Temperament, Attachment Style, and the Psychological Impact of the COVID-19 Outbreak: An Early Report on the Italian General Population. Brain. Behav. Immun..

[9] Salfi F., Lauriola M., Amicucci G., Corigliano D., Viselli L., Tempesta D., Ferrara M. (2020). Gender-Related Time Course of Sleep Disturbances and Psychological Symptoms during the COVID-19 Lockdown: A Longitudinal Study on the Italian Population. Neurobiol. Stress.

[10] Ballesio A., Zagaria A., Musetti A., Lenzo V., Palagini L., Quattropani M.C., Vegni E., Bonazza F., Filosa M., Manari T. (2022). Longitudinal Associations between Stress and Sleep Disturbances during COVID-19. Stress Health.

[11] Holzinger B., Mayer L., Nierwetberg F., Klösch G. (2021). COVID-19 Lockdown—Are Austrians Finally Able to Compensate Their Sleep Debt?. Sleep Med. X.

[12] Cellini N., Canale N., Mioni G., Costa S. (2020). Changes in Sleep Pattern, Sense of Time and Digital Media Use during COVID-19 Lockdown in Italy. J. Sleep Res..

[13] Kocevska D., Blanken T.F., Van Someren E.J.W., Rösler L. (2020). Sleep Quality during the COVID-19 Pandemic: Not One Size Fits All. Sleep Med..

[14] Wright K.P., Linton S.K., Withrow D., Casiraghi L., Lanza S.M., de la Iglesia H., Vetter C., Depner C.M. (2020). Sleep in University Students Prior to and during COVID-19 Stay-at-Home Orders. Curr. Biol..

[15] Salfi F., Amicucci G., Corigliano D., D’Atri A., Viselli L., Tempesta D., Ferrara M. (2021). Changes of Evening Exposure to Electronic Devices during the COVID-19 Lockdown Affect the Time Course of Sleep Disturbances. Sleep.

[16] Salfi F., D’Atri A., Tempesta D., Ferrara M. (2021). Sleeping under the Waves: A Longitudinal Study across the Contagion Peaks of the COVID-19 Pandemic in Italy. J. Sleep Res..

[17] Massar S.A.A., Ng A.S.C., Soon C.S., Ong J.L., Chua X.Y., Chee N.I.Y.N., Lee T.S., Chee M.W.L. (2022). Reopening after Lockdown: The Influence of Working-from-Home and Digital Device Use on Sleep, Physical Activity, and Wellbeing Following COVID-19 Lockdown and Reopening. Sleep.

[18] Gorgoni M., Scarpelli S., Alfonsi V., De Gennaro L. (2022). Dreaming during the COVID-19 Pandemic: A Narrative Review. Neurosci. Biobehav. Rev..

[19] Fränkl E., Scarpelli S., Nadorff M.R., Bjorvatn B., Bolstad C.J., Chan N.Y., Chung F., Dauvilliers Y., Espie C.A., Inoue Y. (2021). How Our Dreams Changed during the COVID-19 Pandemic: Effects and Correlates of Dream Recall Frequency—A Multinational Study on 19,355 Adults. Nat. Sci. Sleep.

[20] Schredl M., Bulkeley K. (2020). Dreaming and the COVID-19 Pandemic: A Survey in a U.S. Sample. Dreaming.

[21] Scarpelli S., Alfonsi V., Mangiaruga A., Musetti A., Quattropani M.C., Lenzo V., Freda M.F., Lemmo D., Vegni E., Borghi L. (2021). Pandemic Nightmares: Effects on Dream Activity of the COVID-19 Lockdown in Italy. J. Sleep Res..

[22] Solomonova E., Picard-Deland C., Rapoport I.L., Pennestri M.H., Saad M., Kendzerska T., Veissiere S.P.L., Godbout R., Edwards J.D., Quilty L. (2021). Stuck in a Lockdown: Dreams, Bad Dreams, Nightmares, and Their Relationship to Stress, Depression and Anxiety during the COVID-19 Pandemic. PLoS ONE.

[23] Scarpelli S., Nadorff M.R., Bjorvatn B., Chung F., Dauvilliers Y., Espie C.A., Inoue Y., Matsui K., Merikanto I., Morin C.M. (2022). Nightmares in People with COVID-19: Did Coronavirus Infect Our Dreams?. Nat. Sci. Sleep.

[24] Gorgoni M., Scarpelli S., Alfonsi V., Annarumma L., Cordone S., Stravolo S., De Gennaro L. (2021). Pandemic Dreams: Quantitative and Qualitative Features of the Oneiric Activity during the Lockdown Due to COVID-19 in Italy. Sleep Med..

[25] Scarpelli S., Alfonsi V., Gorgoni M., Musetti A., Filosa M., Quattropani M.C., Lenzo V., Vegni E., Borghi L., Margherita G. (2021). Dreams and Nightmares during the First and Second Wave of the COVID-19 Infection: A Longitudinal Study. Brain Sci..

[26] Scarpelli S., Alfonsi V., Camaioni M., Gorgoni M., Albano A., Musetti A., Quattropani M.C., Plazzi G., De Gennaro L., Franceschini C. (2023). Longitudinal Findings on the Oneiric Activity Changes Across the Pandemic. Nat. Sci. Sleep.

[27] Remedios A., Marin-Dragu S., Routledge F., Hamm S., Iyer R.S., Orr M., Meier S., Michael S. (2023). Nightmare Frequency and Nightmare Distress during the COVID-19 Pandemic. J. Clin. Sleep Med..

[28] Pesonen A.K., Lipsanen J., Halonen R., Elovainio M., Sandman N., Mäkelä J.M., Antila M., Béchard D., Ollila H.M., Kuula L. (2020). Pandemic Dreams: Network Analysis of Dream Content During the COVID-19 Lockdown. Front. Psychol..

[29] Montplasir J., Zadra A., Nielsen T., Petit D. (2017). Basic Science, Technical Considerations and Clinical Aspects. Sleep Disorders Medicine.

[30] Liu Y., Partinen E., Chan N.Y., Dauvilliers Y., Inoue Y., De Gennaro L., Plazzi G., Bolstad C.J., Nadorff M.R., Merikanto I. (2023). Dream-Enactment Behaviours during the COVID-19 Pandemic: An International COVID-19 Sleep Study. J. Sleep Res..

[31] Koulack D., Goodenough D.R. (1976). Dream Recall and Dream Recall Failure: An Arousal-Retrieval Model. Psychol. Bull..

[32] van Wyk M., Solms M., Lipinska G. (2019). Increased Awakenings from Non-Rapid Eye Movement Sleep Explain Differences in Dream Recall Frequency in Healthy Individuals. Front. Hum. Neurosci..

[33] Alfonsi V., D’Atri A., Scarpelli S., Mangiaruga A., De Gennaro L. (2019). Sleep Talking: A Viable Access to Mental Processes during Sleep. Sleep Med. Rev..

[34] Mangiaruga A., D’Atri A., Scarpelli S., Alfonsi V., Camaioni M., Annarumma L., Gorgoni M., Pazzaglia M., De Gennaro L. (2022). Sleep Talking versus Sleep Moaning: Electrophysiological Patterns Preceding Linguistic Vocalizations during Sleep. Sleep.

[35] Camaioni M., Scarpelli S., Alfonsi V., Gorgoni M., De Bartolo M., Calzolari R., De Gennaro L. (2022). The Influence of Sleep Talking on Nocturnal Sleep and Sleep-Dependent Cognitive Processes. J. Clin. Med..

[36] Hublin C., Kaprio J., Partinen M., Koskenvuo M. (2001). Parasomnias: Co-Occurrence and Genetics. Psychiatr. Genet..

[37] Klackenberg G., Guilleminault C. (1987). Incidence of Parasomnias in Children in a General Population. Sleep and Its Disorders in Children.

[38] Curcio G., Tempesta D., Scarlata S., Marzano C., Moroni F., Rossini P.M., Ferrara M., De Gennaro L. (2013). Validity of the Italian Version of the Pittsburgh Sleep Quality Index (PSQI). Neurol. Sci..

[39] Fulda S., Hornyak M., Müller K., Cerny L., Beitinger P.A., Wetter T.C. (2008). Development and Validation of the Munich Parasomnia Screening (MUPS): A Questionnaire for Parasomnias and Nocturnal Behaviors. Somnologie.

[40] De Gennaro L., Ferrara M., Cristiani R., Curcio G., Martiradonna V., Bertini M. (2003). Alexithymia and Dream Recall upon Spontaneous Morning Awakening. Psychosom. Med..

[41] Casagrande M., Cortini P. (2008). Spoken and Written Dream Communication: Differences and Methodological Aspects. Conscious. Cogn..

[42] De Gennaro L., Cipolli C., Cherubini A., Assogna F., Cacciari C., Marzano C., Curcio G., Ferrara M., Caltagirone C., Spalletta G. (2010). Amygdala and Hippocampus Volumetry and Diffusivity in Relation to Dreaming. Hum. Brain Mapp..

[43] Lehmann S., Skogen J.C., Haug E., Mæland S., Fadnes L.T., Sandal G.M., Hysing M., Bjørknes R. (2021). Perceived Consequences and Worries among Youth in Norway during the COVID-19 Pandemic Lockdown. Scand. J. Public Health.

[44] Ohayon M.M., Morselli P.L., Guilleminault C. (1997). Prevalence of Nightmares and Their Relationship to Psychopathology and Daytime Functioning in Insomnia Subjects. Sleep.

[45] Berlin R.M., Litovitz G.L., Diaz M.A., Ahmed S.W. (1984). Sleep Disorders on a Psychiatric Consultation Service. Am. J. Psychiatry.

[46] Cernovsky Z.Z., Paitich D., Crawford G. (1986). MMPI and Nightmare Reports in Women Addicted to Alcohol and Other Drugs. Percept. Mot. Skills.

[47] Munezawa T., Kaneita Y., Osaki Y., Kanda H., Ohtsu T., Suzuki H., Minowa M., Suzuki K., Higuchi S., Mori J. (2011). Nightmare and Sleep Paralysis among Japanese Adolescents: A Nationwide Representative Survey. Sleep Med..

[48] Tanskanen A., Tuomilehto J., Viinamäki H., Vartiainen E., Lehtonen J., Puska P. (2001). Nightmares as Predictors of Suicide. Sleep.

[49] Kales A., Soldatos C.R., Caldwell A.B., Charney D.S., Kales J.D., Markel D., Cadieux R. (1980). Nightmares: Clinical Characteristics and Personality Patterns. Am. J. Psychiatry.

[50] Kramer M., Schoen L.S., Kinney L. (1984). Psychological and Behavioral Features of Disturbed Dreamers. Psychiatr. J. Univ. Ott..

[51] Berquier A., Ashton R. (1992). Characteristics of the Frequent Nightmare Sufferer. J. Abnorm. Psychol..

[52] Husni M., Cernovsky Z.Z., Koye N., Haggarty J. (2001). Nightmares of Refugees from Kurdistan. J. Nerv. Ment. Dis..

[53] Takeuchi T., Fukuda K., Sasaki Y., Inugami M., Murphy T.I. (2002). Factors Related to the Occurrence of Isolated Sleep Paralysis Elicited during a Multi-Phasic Sleep-Wake Schedule. Sleep.

[54] Hinton D.E., Pich V., Chhean D., Pollack M.H., McNally R.J. (2005). Sleep Paralysis among Cambodian Refugees: Association with PTSD Diagnosis and Severity. Depress. Anxiety.

[55] Ohayon M.M., Shapiro C.M. (2000). Sleep Disturbances and Psychiatric Disorders Associated with Posttraumatic Stress Disorder in the General Population. Compr. Psychiatry.

[56] Yeung A., Xu Y., Chang D.F. (2005). Prevalence and Illness Beliefs of Sleep Paralysis among Chinese Psychiatric Patients in China and the United States. Transcult. Psychiatry.

[57] Castelnovo A., Lopez R., Proserpio P., Nobili L., Dauvilliers Y. (2018). NREM Sleep Parasomnias as Disorders of Sleep-State Dissociation. Nat. Rev. Neurol..

[58] Morin C.M., Bjorvatn B., Chung F., Holzinger B., Partinen M., Penzel T., Ivers H., Wing Y.K., Chan N.Y., Merikanto I. (2021). Insomnia, Anxiety, and Depression during the COVID-19 Pandemic: An International Collaborative Study. Sleep Med..

[59] Salari N., Hosseinian-Far A., Jalali R., Vaisi-Raygani A., Rasoulpoor S., Mohammadi M., Rasoulpoor S., Khaledi-Paveh B. (2020). Prevalence of Stress, Anxiety, Depression among the General Population during the COVID-19 Pandemic: A Systematic Review and Meta-Analysis. Global. Health.

[60] Barone D.A., Henchcliffe C. (2018). Rapid Eye Movement Sleep Behavior Disorder and the Link to Alpha-Synucleinopathies. Clin. Neurophysiol..

[61] Scarpelli S., Alfonsi V., Gorgoni M., De Gennaro L. (2022). What about Dreams? State of the Art and Open Questions. J. Sleep Res..

[62] Scarpelli S., Bartolacci C., D’Atri A., Gorgoni M., De Gennaro L. (2019). The Functional Role of Dreaming in Emotional Processes. Front. Psychol..

[63] Rechtschaffen A., Goodenough D.R., Shapiro A. (1962). Patterns of Sleep Talking. Arch. Gen. Psychiatry.

[64] Castelnovo A., Loddo G., Provini F., Miano S., Manconi M. (2021). Mental Activity during Episodes of Sleepwalking, Night Terrors or Confusional Arousals: Differences between Children and Adults. Nat. Sci. Sleep.

[65] Fasiello E., Scarpelli S., Gorgoni M., Alfonsi V., Galbiati A., De Gennaro L. (2023). A Systematic Review of Dreams and Nightmares Recall in Patients with Rapid Eye Movement Sleep Behaviour Disorder. J. Sleep Res..

[66] Uguccioni G., Golmard J.-L., de Fontreaux A.N., Leu-Semenescu S., Brion A., Arnulf I. (2013). Fight or Flight? Dream Content during Sleepwalking/Sleep Terrors vs. Rapid Eye Movement Sleep Behavior Disorder. Sleep Med..

[67] Antrobus J. (1991). Dreaming: Cognitive Processes During Cortical Activation and High Afferent Thresholds. Psychol. Rev..

[68] Casagrande M., Violani C., Lucidi F., Buttinelli E., Bertini M. (1996). Variations in Sleep Mentation as a Function of Time of Night. Int. J. Neurosci..

[69] Foulkes D. (1967). Nonrapid Eye Movement Mentation. Exp. Neurol..

[70] Rechtschaffen A., Verdone P., Wheaton J. (1963). Reports of mental activity during sleep. Can. Psychiatr. Assoc. J..

[71] MacNeilage P.F. (1970). Motor Control of Serial Ordering of Speech. Psychol. Rev..

[72] Arkin A.M., Toth F., Baker J., Hastey J.M. (1970). The Frequency of Sleep Talking in the Laboratory among Chronic Sleep Talkers and Good Dream Recallers. J. Nerv. Ment. Dis..

